# The genomic and transcriptomic landscape of anaplastic thyroid cancer: implications for therapy

**DOI:** 10.1186/s12885-015-1955-9

**Published:** 2015-12-18

**Authors:** Katayoon Kasaian, Sam M. Wiseman, Blair A. Walker, Jacqueline E. Schein, Yongjun Zhao, Martin Hirst, Richard A. Moore, Andrew J. Mungall, Marco A. Marra, Steven JM Jones

**Affiliations:** Canada’s Michael Smith Genome Sciences Centre, British Columbia Cancer Agency, Vancouver, British Columbia Canada; Department of Surgery, St. Paul’s Hospital and University of British Columbia, Vancouver, British Columbia Canada; Department of Pathology and Laboratory Medicine, St. Paul’s Hospital and University of British Columbia, Vancouver, British Columbia Canada; Department of Medical Genetics, University of British Columbia, Vancouver, British Columbia Canada; Department of Molecular Biology and Biochemistry, Simon Fraser University, Burnaby, British Columbia Canada; 570 West 7th Ave, Vancouver, British Columbia V5Z 4S6 Canada

**Keywords:** Anaplastic thyroid carcinoma, cell line, whole genome and transcriptome sequencing, *FGFR2-OGDH* fusion, *SS18-SLC5A11* fusion, *MKRN1-BRAF* fusion, epigenetic alterations, mTOR signaling pathway, therapy targets

## Abstract

**Background:**

Anaplastic thyroid carcinoma is the most undifferentiated form of thyroid cancer and one of the deadliest of all adult solid malignancies. Here we report the first genomic and transcriptomic profile of anaplastic thyroid cancer including those of several unique cell lines and outline novel potential drivers of malignancy and targets of therapy.

**Methods:**

We describe whole genomic and transcriptomic profiles of 1 primary anaplastic thyroid tumor and 3 authenticated cell lines. Those profiles augmented by the transcriptomes of 4 additional and unique cell lines were compared to 58 pairs of papillary thyroid carcinoma and matched normal tissue transcriptomes from The Cancer Genome Atlas study.

**Results:**

The most prevalent mutations were those of *TP53* and *BRAF*; repeated alterations of the epigenetic machinery such as frame-shift deletions of *HDAC10* and *EP300*, loss of *SMARCA2* and fusions of *MECP2*, *BCL11A* and *SS18* were observed. Sequence data displayed aneuploidy and large regions of copy loss and gain in all genomes. Common regions of gain were however evident encompassing chromosomes 5p and 20q. We found novel anaplastic gene fusions including *MKRN1-BRAF*, *FGFR2-OGDH* and *SS18-SLC5A11*, all expressed in-frame fusions involving a known proto-oncogene. Comparison of the anaplastic thyroid cancer expression datasets with the papillary thyroid cancer and normal thyroid tissue transcriptomes suggested several known drug targets such as *FGFRs*, *VEGFRs*, *KIT* and *RET* to have lower expression levels in anaplastic specimens compared with both papillary thyroid cancers and normal tissues, confirming the observed lack of response to therapies targeting these pathways. Further integrative data analysis identified the mTOR signaling pathway as a potential therapeutic target in this disease.

**Conclusions:**

Anaplastic thyroid carcinoma possessed heterogeneous and unique profiles revealing the significance of detailed molecular profiling of individual tumors and the treatment of each as a unique entity; the cell line sequence data promises to facilitate the more accurate and intentional drug screening studies for anaplastic thyroid cancer.

**Electronic supplementary material:**

The online version of this article (doi:10.1186/s12885-015-1955-9) contains supplementary material, which is available to authorized users.

## Background

Anaplastic thyroid carcinoma (ATC) is an uncommon malignancy that accounts for only 1-2 % of thyroid cancers and yet it is responsible for 14-39 % of all thyroid cancer related deaths [1, 2]. Dedifferentiation of thyroid follicular cells in the course of tumor evolution results in this most aggressive form of thyroid cancer and one of the deadliest of all adult solid malignancies with 68.4 % and 80.7 % mortality rates at 6 and 12 moths, respectively [2]. A study of 516 patients from 12 population-based cancer registries recorded in the Surveillance, Epidemiology and End Results database between 1973 and 2000 found that diagnosis made before the age of 60, confined disease to the thyroid and treatment with surgical resection and external beam radiation therapy are associated with better, but still dismal, survival in ATC patients [2]. Though aggressive multimodal treatment strategies may achieve better survival for those patients who present with fewer disease risks, for those with worse prognosis and extensive local and distant involvement at diagnosis, such treatments could worsen quality of life [3]. No effective or standard therapy for the treatment of anaplastic thyroid cancer exists; several clinical trials involving a small number of patients have failed to demonstrate any prolonged response and the use of chemotherapeutics such as doxorubicin and paclitaxel has not shown any significant survival benefits [2, 3]. Multikinase inhibitors have more recently been used in the treatment of advanced and refractory thyroid cancers, and although some of these result in objective responses and can improve survival in select patients with differentiated thyroid cancers (DTC), the response of ATCs has been less consequential [1].

The rare occurrence of ATC and the rapid death and short follow-ups as a result of its aggressive progression have made it challenging to study the biology of the disease or to conduct clinical trials where responses to novel therapies can be examined [4]. Retrospective studies of small cohorts of patients have found anaplastic thyroid carcinoma to be a heterogeneous disease on the molecular level, rendering it impossible to define a common and specific route of oncogenic transformation and thus to identify effective therapeutics [5]. Mutations of various pathways including MAPK, PI3K and Wnt have been described as potential drivers of this malignancy [5, 6]. A recent whole exome sequencing experiment also identified repeated alterations of MAPK, ErbB and RAS signaling pathways and described mutations in genes not previously implicated in ATC such as *mTOR*, *NF1*, *NF2*, *MLH1*, *MLH3*, *MSH5, MSH6, ERBB2, EIF1AX* and *USH2A* [7]. Alterations of MAPK and PI3K pathways are shared with the less lethal DTCs, suggesting their progression to ATC through step-wise accumulation of mutations and tumor evolution [4]; however, dedifferentiation of preexisting benign nodules and DTCs are not the only means of disease development and at least a subset of ATCs may arise *de novo* [5].

Tumor-derived cell lines provide an alternative to studying patient specimens when profiling rare tumors and these can facilitate the investigation of therapeutic effectiveness in pre-clinical settings. Schweppe and colleagues have reported on cross-contamination and mislabeling concerns in 40 % of thyroid cancer cell lines that have been used in over 200 published studies [8, 9]. They have clearly emphasized the need for detailed characterization of all thyroid-derived, including ATC-derived, cell lines. In this study, we describe the genomic and transcriptomic profiles of 1 primary ATC and 3 authenticated anaplastic thyroid cancer cell lines [9]. Those profiles augmented by the transcriptomes of 4 additional and unique cell lines [8] were compared to 58 pairs of papillary thyroid carcinoma (PTC) and matched normal tissue transcriptomes from The Cancer Genome Atlas (TCGA) study [10]. To the best of our knowledge, this is the first report of whole genome and transcriptome analyses of anaplastic thyroid cancer, allowing for the identification of regions of copy number alteration and large structural events at the base level resolution.

## Methods

### Study specimens

Excision biopsy of a primary and treatment-naive anaplastic thyroid carcinoma tumor and peripheral blood sample were collected from a 63-year old male at the time of palliative thyroidectomy; the patient lacking prior personal or family history of thyroid disease or cancer and radiation exposure presented with lung metastasis. He provided written informed consent for the complete genomic profiling of his specimens; these were collected as part of a research project approved by the British Columbia Cancer Agency’s Research Ethics Board and are in accordance with the Declaration of Helsinki. In addition, 3 authenticated ATC cell lines, THJ-16T, THJ-21T and THJ-29T [9], obtained from the Mayo Clinic (Jacksonville, FL) and 4 unique cell lines [8], ACT-1 and T238 from Dr. R. Schweppe at the University of Colorado (Denver, Colorado) and C643 and HTh7 from Dr. N.E. Heldin at the Karolinska Institute (Uppsala, Sweden), were evaluated in this study.

### Library preparation and sequencing

DNA from the ATC tumor, the matched peripheral blood specimen, and THJ-16T, THJ-21T and THJ-29T cell lines were subjected to whole genome sequencing; 100 bp paired-end sequence reads were generated on Illumina HiSeq2500 instruments following the manufacturer’s protocol with minor variations. In addition, 75 bp paired-end transcriptome sequence reads were produced for the tumor and all 7 cell lines. The aligned sequence datasets have been deposited at the protected European Genome-phenome Archive (EGA, http://www.ebi.ac.uk/ega/) under accession number EGAS00001001214. Library construction and sequencing protocols are detailed in the supplementary material.

### Sequence data analysis

Sequence reads from the whole genome libraries were aligned to the human reference genome (build GRCh37) using the Burrows-Wheeler Alignment (BWA) tool [11]. The tumor’s genomic sequence was compared to that of patient’s constitutive DNA to identify somatic alterations. Regions of copy number variation (CNV) and loss of heterozygosity (LOH) were determined using Control-FREEC [12]. *De novo* assembly and annotation of genomic data using ABySS and Trans-ABySS [13] were used to identify small insertions and deletions (indels) and larger structural variants (SVs) including translocations, inversion and duplications leading to gene fusions; identified SVs were verified using an orthogonal alignment-based detection tool, BreakDancer [14]. Single nucleotide variants (SNVs) and indels in the tumor/normal pair were identified using a probabilistic joint variant calling approach utilizing SAMtools and Strelka [15, 16]. Variants in the unpaired cell line genomic data were identified using SAMtools [15]; the indel lists for these samples were refined to include only those events that were also called through *de novo* assembly.

Sequence reads from the transcriptome libraries were aligned to the human reference genome (build GRCh37) using TopHat [17] with Ensembl gene model annotation file on the -G parameter. The reference sequence and the corresponding annotation files were provided by Illumina’s iGenome project and downloaded from the TopHat homepage (https://ccb.jhu.edu/software/tophat/igenomes.shtml). Quantification of gene expression was accomplished using HTSeq [18] in intersection-nonempty mode and excluding reads with quality less than 10, all subsequent analyses were run using only the count values for the protein-coding elements. Fifty-eight pairs of papillary thyroid carcinoma and matched normal tissue transcriptomes from The Cancer Genome Atlas project [10] were used for differential gene expression analysis. To ensure consistent analysis, raw sequence reads were downloaded from the Cancer Genome Hub and processed using the analysis pipeline described above. Protein-coding gene read counts were used as input into the R package edgeR [19] for differential gene expression analysis. Single-sample gene set enrichment analysis (ssGSEA) [20] was performed for each of the 8 transcriptomes to elucidate the oncogenic profiles enriched in each library when compared with normal thyroid tissue expression profiles. Structural variants were identified using *de novo* assembly-based approach employing ABySS and Trans-ABySS [13] and the alignment-based SV detection tool Minimum Overlap Junction Optimizer (MOJO) (https://github.com/cband/MOJO).

## Results

### Single nucleotide variants and indels

Twenty-four somatic SNVs and indels were identified in the tumor’s genome including heterozygous BRAF p.V600E and TP53 p.Y163C mutations. All three cell lines had *TP53* homozygous nonsense or missense mutations with known pathogenic alleles. Other variants related to tumor biology included a homozygous BRAF p.V600E mutation in THJ-21T and heterozygous and homozygous frame-shift deletions of HDAC10 (p.H134Tfs) and CDKN2A (p.Q70Sfs), respectively, in THJ-29T. Additionally, THJ-16T harbored a heterozygous activating mutation in PIK3CA (p.E545K), a variant of unknown significance in RET (p.E90K) and a homozygous frame-shift deletion (p.S799Ffs) in EP300. Alterations of *TP53* and *BRAF* were the only recurrent events and no mutations of the previously described ATC genes including *H-*, *K-*, *N-RAS*, *CTNNB1*, *IDH1*, *ALK*, *PTEN*, *APC*, or *AXIN1* [6, 7, 21] were identified in these specimens. This is likely due to a small number of samples examined here and the infrequent mutations of these genes in the overall ATC population [6]. All identified protein-coding variants are listed in the Additional file 1.

### Copy number variants

**Fig. 1:**
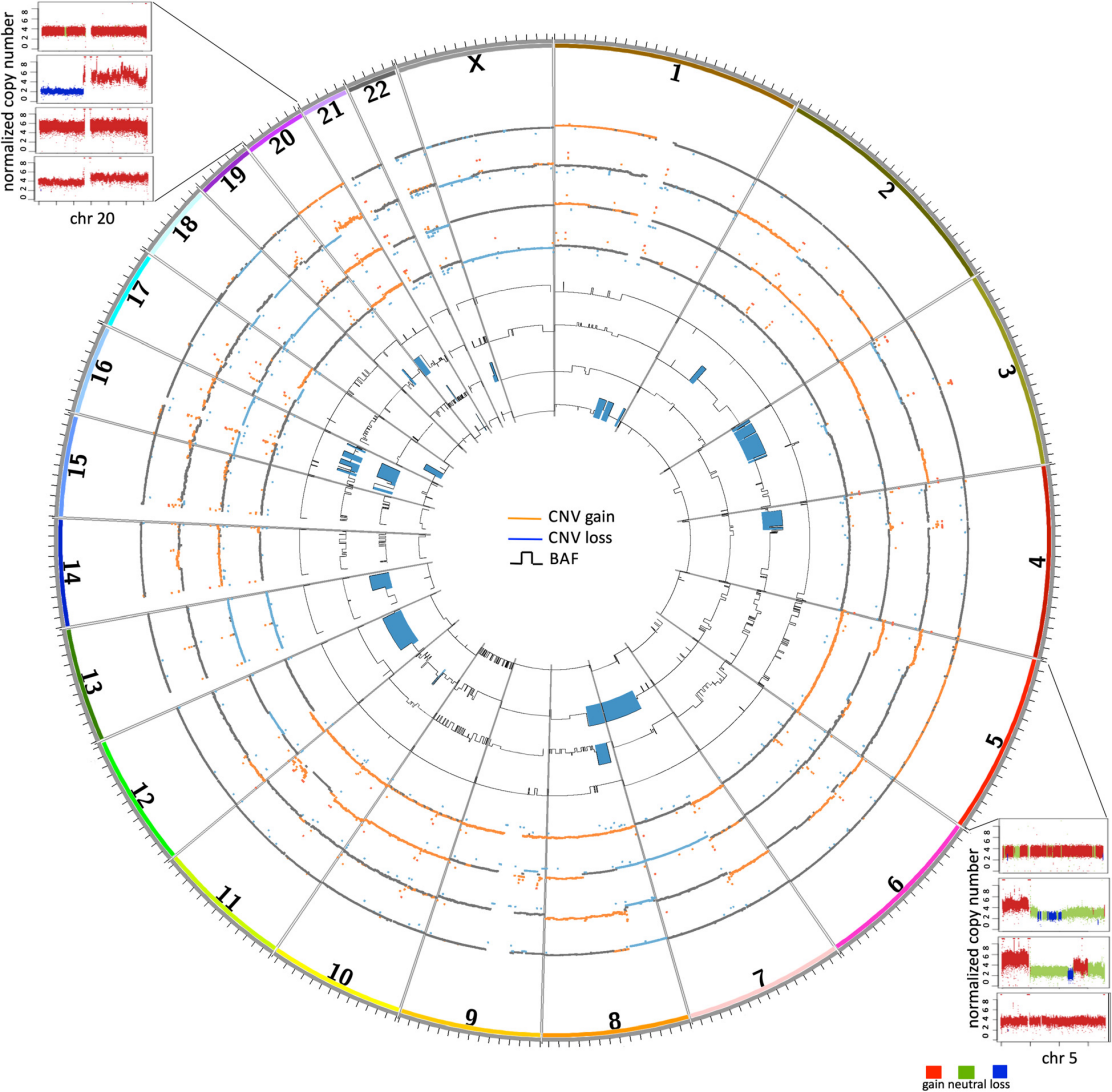
Regions of copy number variation and loss of heterozygosity. A circos plot depicting, from the outer ring inward, tumor CNV, THJ-29T CNV, THJ-21T CNV, THJ-16T CNV, tumor LOH, THJ-29T LOH, THJ-21T LOH and THJ-16T LOH. Red and blue CNV regions illustrate the regions of copy gain and loss, respectively. The LOH tracks illustrate the B Allele Frequencies (BAF) ranging from 0.5 to 1. Those regions with BAF > = 0.9 are highlighted in blue. Regions of 5p and 20q showed recurrent copy gain in all samples

Evaluation of the copy number status and single nucleotide allele frequencies of the genomic data revealed extensive regions of gene copy loss and gain and the presence of triploid genomes in all 4 samples (Fig. 1), consistent with previous observations of aneuploidy in the majority of ATCs [22]. Large-scale copy number changes have also been described in ATCs [1] and are a hallmark of the progression from the mostly “quiet” differentiated cancers [10] to the aggressive and lethal ATCs. Although the tumor and the cell lines showed variable regions of copy number alterations, a 26 Mb minimal region on 5p, encompassing 196 genes, and the long arm of chromosome 20 showed gain of extra gene copies in all samples (Fig. 1). High-level and recurrent amplifications of 5p and chromosome 20 have been reported in studies utilizing comparative genomic hybridization in studying ATCs [21] indicating that genes located in these regions might play an important role in ATC tumor initiation and/or progression. The 5p region includes proto-oncogenes such as *FGF10* and *SKP2*, mTOR signaling pathway members *RICTOR* and *PRKAA1*, in addition to *IL7R*, *OSMR*, *LIFR*, *PRLR* and *GHR*, all receptors involved in JAK-STAT and the downstream PI3K-Akt pathways. Anti-apoptotic and cell cycle genes *BCL2L1*, *YWHAB*, *E2F1* and *AURKA*, proto-oncogenes *PLCG1* and *STK4* and chromatin remodeling genes *ASXL1*, *CHD6* and *DNMT3B* have all gained extra copies through the amplification of 20q. Noteworthy observations of copy number change included the presence of 15 copies of each of *KDR/VEGFR1*, *KIT* and *PDGFRA* in a region of focal amplification on chromosome 4 in THJ-29T cell line. THJ-21T showed a region of high amplification on chromosome 11 leading to the accumulation of 25 copies of each of *BIRC2*, *BIRC3*, *MMP1*/*3*/*7*/*8*/*10*/*13*/*27* and *YAP1*; this cell line also had a complete loss of a small region on chromosome 9 encompassing *SMARCA2*, a member of the SWI/SNF complex, and *GLIS3*, a transcription factor implicated in the development and normal functioning of the thyroid (Additional file 2: Figure S1). Protein-coding genes with changes in copy number and their referred copy numbers from the sequence data are listed in the Additional file 1.

### Structural variants

**Fig. 2:**
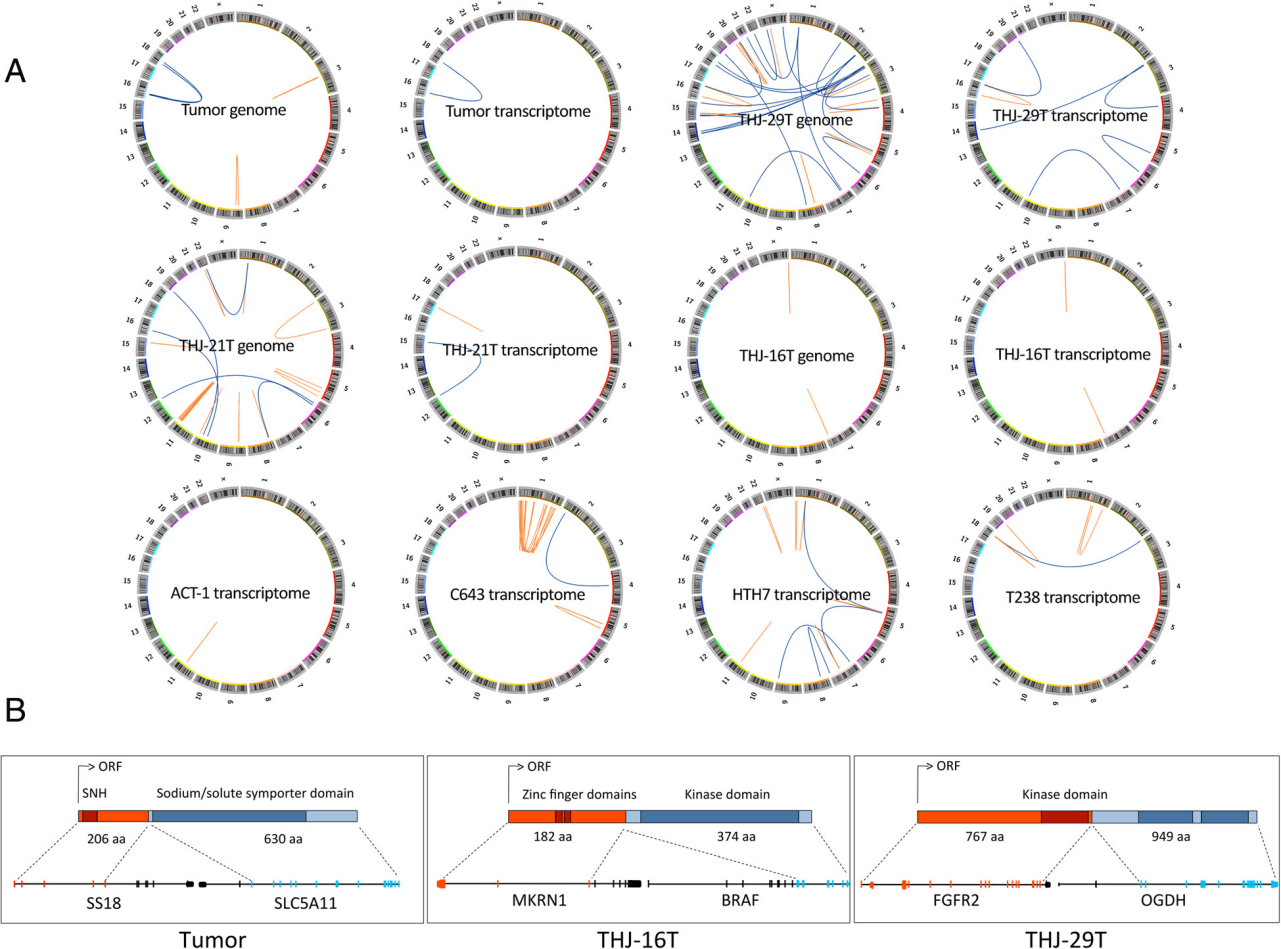
Somatic structural variants in ATC genomes and transcriptomes **a.** Structural variants identified in the genomic and transcriptomic datasets **b.** Detailed structure of the potentially oncogenic fusions: *SS18* (transcript: ENST00000415083)/*SLC5A11* (transcript: ENST00000347898) fusion in the tumor, *MKRN1* (transcript: ENST00000255977)/*BRAF* (transcript: ENST00000288602) fusion in THJ-16T cell line and *FGFR2* (transcript: ENST00000358487)/*OGDH* (transcript: ENST00000222673) fusion in THJ-29T cell line

The study specimens were found to have anywhere between 1 to 32 structural variants (Fig. 2a and Additional file 1). Expressed in-frame gene fusions involving at least one proto-oncogene have been described in various cancers and are shown to be the driver of malignant phenotype, at times as the only such event in the tumor. We identified instances of these fusions in the genomes of THJ-16T and THJ-29T cell lines and the tumor (Fig. 2b). These included an MKRN1-BRAF fusion in THJ-16T; the fusion product has lost the N terminal regulatory region of BRAF while retaining its kinase domain, hence likely leading to the constitutive activation of the kinase. A fusion of these two genes was also found in 1 TCGA PTC sample (0.2 % population frequency) [10]. A reciprocal fusion between chromosomes 7 and 10 led to an in-frame fusion of FGFR2 and OGDH in THJ-29T, retaining the growth factor receptor’s kinase domain. Two TCGA PTC cases were also reported to have *FGFR2* gene fusions with *VCL* and *OFD1* as partners [10]. *FGFR2* is found fused to various genes in different cancers where the fusion partners facilitate its constitutive activation through providing dimerization domains [23]. Sensitivity to FGFR inhibitors have been observed in patients harboring *FGFR2* fusions with the same breakpoint as that found in the THJ-29T ATC cell line [23] and thus testing for these fusions might provide a tractable therapeutic option for a subset of patients diagnosed with anaplastic thyroid cancer. We also identified a translocation between chromosomes 16 and 18 in the tumor, fusing the proto-oncogene *SS18* and *SLC5A11*. SS18 (also known as SYT) is commonly found fused to one of SSX1, SSX2 or SSX4 in synovial sarcomas [24]. In addition to the above potentially oncogenic fusions, gene members of the axon guidance pathway, recurrently altered in pancreatic cancer [25], were also found to be involved in multiple fusions: *CADM2-EPHA3* fusion in the tumor’s genome, fusion of chromosome 19 to *SLIT1* on chromosome 10 in the THJ-21T genome and *SRGAP3-SETD5* fusion in THJ-29T (Additional file 1).

### Analysis of differential transcript abundance

**Fig. 3:**
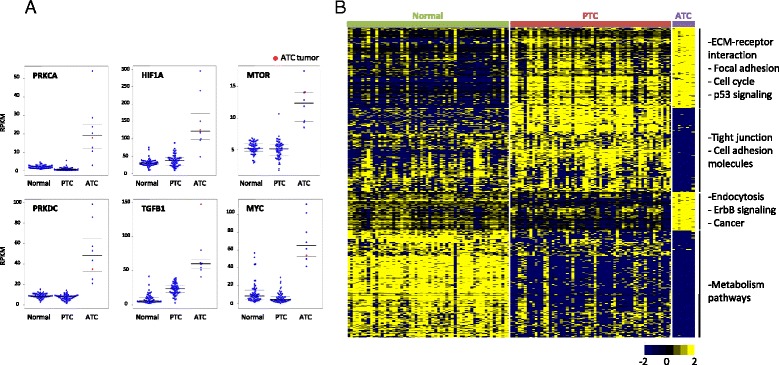
Transcriptomic analysis of ATCs a. The expression levels (RPKM = reads per kilobase per million mapped reads) of select genes in the TCGA and ATC specimens are plotted. Median, first and third quartile values are marked for each distribution **b.** Samples were ordered on the basis of pathology and 1647 significantly expressed genes in 58 TCGA normal thyroid tissue transcriptomes, 58 TCGA papillary thyroid cancer transcriptomes and 8 anaplastic thyroid cancer transcriptomes were clustered

**Fig. 4:**
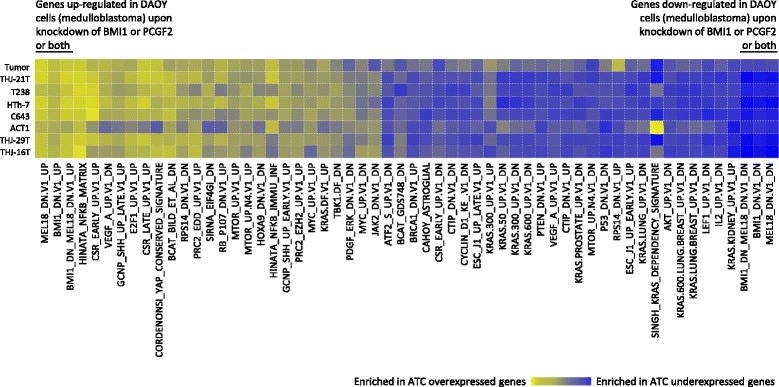
Single-sample gene set enrichment analysis (ssGSEA). ssGSEA was performed for all 8 transcriptome libraries using fold changes in expression of each gene (ATC expression/average expression in 58 normal libraries) in order to identify enriched oncogenic signatures. Top 20 % most enriched signatures that were shared in two or more libraries are plotted. The molecular signatures enriched with up- and down-regulated ATC genes included genes that were up- and down-regulated upon knockdown of *BMI1* or *PCGF2* or both genes [26]. Standard names of the oncogenic signature gene sets from the MSigDB are listed below the plot

Despite the heterogeneous molecular profile of ATCs evident from the lack of commonly mutated genes and oncogenic fusions, the transcriptomic analysis of the tumor and all 7 cell lines showed consistent up- and down-regulation of several genes when compared to the compendium of normal thyroid tissue transcriptomes. Overexpressed genes included focal adhesion, cytoskeleton and ECM-receptor interaction pathway genes such as *ITGA3*, *ITGB1*, *FLNA*, *ACTN1*, and *CD44* indicating alterations of genes involved in regulation of normal cell shape and migration. Cancer-related genes with significant up-regulation in all ATCs included *MYC*, *mTOR*, *PRKCA* and *TGFB1* (Fig. 3a). The down-regulated genes included thyroid differentiation signature genes such as *TG*, *TTF1*, *TSHR* and *TPO* (Additional file 2: Figure S2) in addition to the tumor suppressor *FHIT*. Genes believed to be cancer drivers and to serve as drug targets in other malignancies showed consistent down-regulation in anaplastic thyroid cancer; these included *ERBB4*, *NTRK2*, *FGF7* and *MAPK10* (Additional file 2: Figure S3). Differential gene expression analysis of the ATC cohort against the TCGA normal transcriptomes using edgeR found 840 and 574 genes to be down- and up-regulated in ATCs, respectively (Benjamini-Hochberg *P*< 0.05 and fold change >4 or <-4); similar analysis yielded 605 and 419 down- and up-regulated genes in ATCs when compared to PTCs. Pathway analysis of these differentially expressed genes showed ECM-receptor interaction, focal adhesion, endocytosis, cell cycle, p53 signaling, ErbB signaling and general cancer pathways to be up regulated in ATCs. Common down-regulated networks included tight junctions, cell adhesion molecules and various metabolism pathways (Fig. 3b). Single-sample gene set enrichment analysis pointed to a potential role of epigenomic deregulation in ATCs where the top signatures enriched with over- and under-expressed ATC genes included genes that were up- and down-regulated, respectively, upon knockdown of *BMI1* or *PCGF2*, both members of the Polycomb group [26] (Fig. 4).

## Discussion

Anaplastic thyroid cancer is an extremely aggressive malignancy with dismal prognosis that has had little change in its 4-month median survival rate over the past 50 years [21]. Similar to the case we genomically profiled, the majority of ATC patients present with a rapidly growing neck mass often causing dyspnea, dysphagia and at times vocal cord paralysis [27]. The extremely poor prognosis of ATC is reflected by the current American Joint Committee on Cancer staging system for thyroid cancer in which individuals with anaplastic histopathology, regardless of extent of disease, are classified as having stage IV disease [2]. There are currently no standard therapies for the treatment of anaplastic thyroid cancer as its rarity and rapidly fatal course have made it difficult to study large cohorts of patients and to conduct randomized clinical trials [28]. Doxorubicin is the most commonly used chemotherapeutic agent for the treatment of progressive and metastatic ATC, but has little impact on survival, with a partial response rate estimated to be 10-30%; if administered in combination with cisplatin, it may have slightly higher efficacy [28, 29]. Multimodal treatments comprised of surgical resection, external beam radiation therapy and systemic therapy have been associated with increased survival in some patients [1] though often only effective in managing uncommonly diagnosed localized ATCs [30]. Individual responses to targeted therapies including multi-kinase inhibitors have been reported [31–33], however, no single agent has shown significant improvement in progression-free survival in the setting of a clinical trial and thus none has gained approval for routine clinical use. Phase II trials of pazopanib [34], imatinib [35], gefitinib [36], axitinib [37] and sorafenib [38, 39] in small patient cohorts showed limited or negligible activity. This is despite some of these agents, such as sorafenib, resulting in objective response and receiving approval for the treatment of advanced DTCs.

The important role of increased endothelial cell proliferation and angiogenesis in thyroid cancer progression and maintenance is well recognized [37], and consequently the majority of the tested compounds are aimed at blocking these signaling pathways. The expression of some of the intended targets of these drugs by our ATC specimens, and the 58 pairs of PTC and normal thyroid tissues, are depicted in the Additional file 2: Figures S2 and S3. The majority of these drug targets, including *FGFR1*, *2*, *3* and *4*, *VEGFR1*, *2* and *3*, *PDGFRA*, *PDGFRB*, *KIT* and *RET*, show similar or lower expression in ATCs compared with both normal tissues and PTCs. The extent of messenger RNA expression might not be an accurate estimate of the protein level in the cell, and over-activation of a kinase is not captured on the transcript level, nonetheless, mRNA is an intermediary information molecule and its amount in the cell serves as a surrogate for protein expression levels. Based on the current differential mRNA expression analysis none of the multi-kinase inhibitors with observed response in DTCs would have an effect on the survival of ATC patients; this is in agreement with the failure of all tested compounds to date and has implications in the development of future clinical trials. Lenvatinib has recently gained approval for the treatment of refractory DTCs, but the first described trial for its use in the treatment of 9 ATC patients showed only a median progression-free survival of 5.5 months [40]. We predict, based on the current study, that lenvatinib would not result in prolonged response in ATCs given the lower expression of all its targets (vascular endothelial growth factor receptors 1,2, and 3, fibroblast growth factor receptors 1, 2, 3 and 4, platelet-derived growth factor receptor alpha, RET and KIT) in ATC specimens (Additional file 2: Figure 3). Generally, inhibitors of growth factors and their receptors appear to have a very limited effect on the survival of ATC patients. A similar lack of inefficacy is also found when using vascular disrupting agents. A single agent trial of the fosbretabulin (also known as combretastatin A-4 phosphate) or its combination use with carboplatin/paclitaxel in a cohort of patients, although showed some clinical activity, had no effect on progression-free survival [41, 42].

Analysis of genomic and transcriptomic datasets in this study allowed for identification of potential new drug targets. *TRIP13* has gained extra copies in all specimens as a result of the 5p gain described above. This gene and its binding partner PRKDC promote non-homologous end joining (NHEJ) in cancer cells resulting in chemoresistance in head and neck malignancies where inhibitors of NHEJ, such as Nu7026, are believed to re-sensitize cells to cisplatin [43]. Both *TRIP13* and *PRKDC* show very high expression in the ATCs we studied and could serve as novel targets for therapy. The mTOR signaling pathway is also a putative target and inhibitors such as everolimus may show efficacy in ATC. Mutations of the pathway genes including *mTOR* and the tumor suppressor *TSC2* have been previously described in ATC [7, 31] and a dramatic and long-lasting response to everolimus in an ATC patient with a truncating mutation in *TSC2* was reported [31]. Though no mutations were identified in the current study, a high level expression of *mTOR* and its downstream effector *HIF1A* was observed, thus raising the possibility for the use of mTOR inhibitors (Fig. 3a). Overexpression of *mTOR* or loss of *TSC2*, its negative regulator, through promoting the transcriptional level of *HIF1A* leads to increased angiogenesis that is sensitive to rapamycin treatment [44]. Given that overexpression of vascular growth factor receptors are not likely to directly lead to increased angiogenesis in ATCs, mTOR signaling emerges as a key angiogenesis driving pathway in this cancer. The effect of everolimus on 5 ATC cell lines including HTh7 and C643 were tested by Papewalis and colleagues [45]. They found that both cell lines responded to therapy with HTh7 exhibiting a much higher sensitivity when compared to known responding lymphoma cell lines. Prior to embarking on clinical trials, further *in vitro* and *in vivo* studies are needed to elucidate the mechanism of response and resistance to targeted therapeutics such as mTOR inhibitors.

Tumor genomes frequently show a vast amount of copy number change and aneuploidy. As these can be the side effect of the altered cell cycle machinery and disease progression rather than its driver(s), all copy number changes may not contribute to changes in gene expression levels. Integrative analysis of CNV and expression datasets thus allowed for the identification of correlated changes of these variations in all 4 specimens. Cell cycle kinase *AURKA* and the transcription factor *E2F1*, both located on chromosome 20 with gain of copies, also showed overexpression providing additional evidence for the deregulation of cell cycle control in ATCs. Overexpression of aurora kinase A is believed to be the cause of vast chromosomal abnormalities in ATCs given its key regulatory role in mitotic cell division, chromosome segregation and cytokinesis through association with centrosomes and the mitotic spindle [5, 30]. Several investigational drugs with inhibitory effect on AURKA are under study and these might serve as promising therapeutics in ATCs. It is however imperative to demonstrate the high expression of these kinases as the driver of malignancy rather than just a by-product of the high rate of cell division in cancers particularly ATCs [27]. Similarly, tissue transglutaminase gene (*TGM2*) has gained extra copies in all samples and also shows overexpression compared with normal thyroid tissue and PTCs. Over-activation of *TGM2* in ATCs correlates with its observed over-expression in pancreatic cancer, another aggressive human malignancy with mortality rates close to 100%. TGM2 over-expression leads to tissue invasion, metastasis and chemotherapeutic resistance in cancers of the pancreas [46] and is shown to protect these cancer cells from autophagy leading to growth advantage and resistance to chemotherapy [46]. TGM2 may as a result serve as a direct drug target where its blockage leads to autophagic cell death.

A successful evolutionary history for cancer requires rapid and dynamic changes in the blueprint of the cell. Through providing a larger pool of possible mutational targets, recurrent hits to specific cellular machineries or pathways, rather than the same gene, can accelerate the success of the cancer in overcoming its host defenses. We found alterations of the epigenetic machinery in all 4 ATC specimens with genome sequence data. A translocation of SS18, a member of SWI/SNF complex [47] in the tumor, homozygous frame-shift deletion in the histone acetyltransferase EP300 and a fusion of methyl CpG binding protein MECP2 and F8 in THJ-16T cell line, complete loss of SMARCA2, another member of the SWI/SNF complex and interacting partner of SS18 [47], in THJ-21T, a heterozygous frame-shift deletion in the histone deacetylase HDAC10 and a gene fusion of the transcriptional repressor and member of the SWI/SNF complex BCL11A [47] and GRIP2 in THJ-29T. SS18 is a subunit of the SWI/SNF complex [47] and hence plays a major role in transcriptional regulation of the cell. It also interacts with various members of chromatin remodeling complexes such as SMARCA2, SMARCA4 [24] and EP300 [48] through its conserved N-terminal SNH domain that is found to be indispensible for the transforming ability of SS18-SSX onco-protein [24]. Although the fusion partner, SLC5A11, is distinct from that observed in sarcomas, it is likely that this fusion has transforming potential in ATCs. Only the last 8 residues of SS18 are deleted in its fusion to SSX genes and the mere deletion of these same 8 amino acids in the absence of a fusion partner was shown to disrupt the normal function of the protein [48]. Loss of SS18 C-terminal might be sufficient for tumorigenesis or that a yet unknown function of SLC5A11 may lead to the malignant transformation. The *FGFR2-OGDH* fusion in THJ-29T is, in addition to the involvement of the growth factor receptor, intriguing considering the role of OGDH in the control of metabolism and cellular epigenetic state. OGDH is a metabolic enzyme of the tricarboxylic acid (TCA) cycle and a subunit of the complex which converts 2-oxoglutarate, product of IDH, to succinate, substrate of SDH. Mutations of IDH1 and IDH2 as well as those in SDH have been observed in numerous cancers and found to cause global epigenetic changes in the tumor [49, 50]. 2-oxoglutarate is required for the normal functioning of chromatin-modifying enzymes such as UTX, JARID1C and TET2 [50] and succinate acts as an inhibitor of DNA and histone demethylases [49]; changes in their cellular concentration as a result of OGDH translocation can in turn alter the epigenomic state of ATC cells. Further evidence for the potential role of epigenomic deregulation in ATC came from single-sample GSEA. Top 20% most enriched oncogenic signatures in each of the 8 transcriptome libraries were identified and those shared in two or more libraries are plotted in Fig. 4. Top signatures enriched with over- and under-expressed ATC genes included genes that were up- and down-regulated, respectively, upon knockdown of *BMI1* or *PCGF2* or both genes [26]. BMI1 and PCGF2 are members of the Polycomb group of transcriptional regulators which control the expression of, among others, genes involved in ECM remodeling, cell adhesion and integrin-mediated signaling pathways [26], all of which demonstrated deregulation in ATCs. It is conceivable that understanding the effect of epigenetic changes in anaplastic thyroid cancer could pave the way for the development and application of novel therapeutics in this aggressive solid tumor. Histone deacetylase inhibitor valproic acid, for instance, increases the effect of both doxorubicin and paclitaxel in ATC cells [21] providing *in vitro* experimental evidence for a driving role of deregulated epigenetic control in ATC.

## Conclusions

In this study, we profiled the molecular alterations of several anaplastic thyroid carcinoma specimens including unique and authenticated ATC cell lines. This study is underpowered in drawing general conclusions for this cancer given the availability of only one primary tumor and the often-observed distinct profiles of cell lines and patient specimens. Given the heterogeneous genomic profiles of these samples and the low frequency of recurrent mutations, studies involving larger cohorts of cases through multi-institutional collaborations are required to identify genes at the “long tail” of the mutational spectrum, and to decipher the underlying biology of the disease. Furthermore, lack of common targetable oncogenic mutations, observed responses to targeted therapies in other cancer types harboring the same aberrations as those found in at least a small subset of ATCs [23], and clinical responses to targeted therapies described in individual ATC patients [31–33] calls for a more genotype-driven approach to diagnosis and treatment of this rare and rapidly fatal cancer. With recent advances in molecular and information technology alike, it is anticipated that sequencing-based clinical tests provide the ability to comprehensively assay the large number of diverse and complex mutational forms that can arise, hence facilitating routine application of precision oncology in the clinic.

## Availability of data and materials

The aligned sequence datasets have been deposited at the protected European Genome-phenome Archive (EGA, http://www.ebi.ac.uk/ega/) under accession number EGAS00001001214.

## Additional files

Additional file 1: Supplementary tables listing the identified mutations, copy number variations and structural events. (XLSX 2590 kb) Additional file 2: Supplementary file outlining the detailed sequencing methodology and additional figures. (DOCX 434 kb)

## Abbreviations

ATC: Anaplastic thyroid carcinoma
BWA: Burrows-Wheeler alignment
CNV: Copy number variation
DTC: Differentiated thyroid cancer
LOH: Loss of heterozygosity
MOJO: Minimum overlap junction optimizer
PTC: Papillary thyroid cancer
SNV: Single nucleotide variant
ssGSEA: Single-sample gene set enrichment analysis
SV: Structural variant
TCA: Tricarboxylic acid
TCGA: The cancer genome atlas

## References

[1] Smallridge RC, Ain KB, Asa SL, Bible KC, Brierley JD, Burman KD (2012). American Thyroid Association guidelines for management of patients with anaplastic thyroid cancer. Thyroid.

[2] Kebebew E, Greenspan FS, Clark OH, Woeber KA, McMillan A (2005). Anaplastic thyroid carcinoma. Treatment outcome and prognostic factors. Cancer.

[3] Sugitani I, Miyauchi A, Sugino K, Okamoto T, Yoshida A, Suzuki S (2012). Prognostic factors and treatment outcomes for anaplastic thyroid carcinoma: ATC Research Consortium of Japan cohort study of 677 patients. World J Surg.

[4] Wiseman SM, Loree TR, Hicks WL, Rigual NR, Winston JS, Tan D (2003). Anaplastic thyroid cancer evolved from papillary carcinoma: demonstration of anaplastic transformation by means of the inter-simple sequence repeat polymerase chain reaction. Arch Otolaryngol Head Neck Surg.

[5] Ragazzi M, Ciarrocchi A, Sancisi V, Gandolfi G, Bisagni A, Piana S (2014). Update on anaplastic thyroid carcinoma: morphological, molecular, and genetic features of the most aggressive thyroid cancer. Int J Endocrinol.

[6] Xing M (2013). Molecular pathogenesis and mechanisms of thyroid cancer. Nat Rev Cancer.

[7] Kunstman JW, Juhlin CC, Goh G, Brown TC, Stenman A, Healy JM, et al. Characterization of the mutational landscape of anaplastic thyroid cancer via whole-exome sequencing. Hum Mol Genet. 2015. 10.1093/hmg/ddu749 PMC4380073 25576899

[8] Schweppe RE, Klopper JP, Korch C, Pugazhenthi U, Benezra M, Knauf JA (2008). Deoxyribonucleic acid profiling analysis of 40 human thyroid cancer cell lines reveals cross-contamination resulting in cell line redundancy and misidentification. J Clin Endocrinol Metab.

[9] Marlow LA, D'Innocenzi J, Zhang Y, Rohl SD, Cooper SJ, Sebo T (2010). Detailed molecular fingerprinting of four new anaplastic thyroid carcinoma cell lines and their use for verification of RhoB as a molecular therapeutic target. J Clin Endocrinol Metab.

[10] Cancer Genome Atlas Research Network (2014). Electronic address: giordano@umich.edu, Cancer Genome Atlas Research Network: Integrated genomic characterization of papillary thyroid carcinoma. Cell.

[11] Li H, Durbin R (2010). Fast and accurate long-read alignment with Burrows-Wheeler transform. Bioinformatics.

[12] Boeva V, Popova T, Bleakley K, Chiche P, Cappo J, Schleiermacher G (2012). Control-FREEC: a tool for assessing copy number and allelic content using next-generation sequencing data. Bioinformatics.

[13] Simpson JT, Wong K, Jackman SD, Schein JE, Jones SJ, Birol I (2009). ABySS: a parallel assembler for short read sequence data. Genome Res.

[14] Chen K, Wallis JW, McLellan MD, Larson DE, Kalicki JM, Pohl CS (2009). BreakDancer: an algorithm for high-resolution mapping of genomic structural variation. Nat Methods.

[15] Li H, Handsaker B, Wysoker A, Fennell T, Ruan J, Homer N (2009). The Sequence Alignment/Map format and SAMtools. Bioinformatics.

[16] Saunders CT, Wong WS, Swamy S, Becq J, Murray LJ, Cheetham RK (2012). Strelka: accurate somatic small-variant calling from sequenced tumor-normal sample pairs. Bioinformatics.

[17] Kim D, Pertea G, Trapnell C, Pimentel H, Kelley R, Salzberg SL (2013). TopHat2: accurate alignment of transcriptomes in the presence of insertions, deletions and gene fusions. Genome Biol.

[18] Anders S, Pyl PT, Huber W (2015). HTSeq--a Python framework to work with high-throughput sequencing data. Bioinformatics.

[19] Robinson MD, McCarthy DJ, Smyth GK (2010). edgeR: a Bioconductor package for differential expression analysis of digital gene expression data. Bioinformatics.

[20] Subramanian A, Tamayo P, Mootha VK, Mukherjee S, Ebert BL, Gillette MA (2005). Gene set enrichment analysis: a knowledge-based approach for interpreting genome-wide expression profiles. Proc Natl Acad Sci U S A.

[21] Smallridge RC, Marlow LA, Copland JA (2009). Anaplastic thyroid cancer: molecular pathogenesis and emerging therapies. Endocr Relat Cancer.

[22] Wiseman SM, Loree TR, Rigual NR, Hicks WL, Douglas WG, Anderson GR (2003). Anaplastic transformation of thyroid cancer: review of clinical, pathologic, and molecular evidence provides new insights into disease biology and future therapy. Head Neck.

[23] Borad MJ, Champion MD, Egan JB, Liang WS, Fonseca R, Bryce AH (2014). Integrated genomic characterization reveals novel, therapeutically relevant drug targets in FGFR and EGFR pathways in sporadic intrahepatic cholangiocarcinoma. PLoS Genet.

[24] Nagai M, Tanaka S, Tsuda M, Endo S, Kato H, Sonobe H (2001). Analysis of transforming activity of human synovial sarcoma-associated chimeric protein SYT-SSX1 bound to chromatin remodeling factor hBRM/hSNF2 alpha. Proc Natl Acad Sci U S A.

[25] Biankin AV, Waddell N, Kassahn KS, Gingras MC, Muthuswamy LB, Johns AL (2012). Pancreatic cancer genomes reveal aberrations in axon guidance pathway genes. Nature.

[26] Wiederschain D, Chen L, Johnson B, Bettano K, Jackson D, Taraszka J (2007). Contribution of polycomb homologues Bmi-1 and Mel-18 to medulloblastoma pathogenesis. Mol Cell Biol.

[27] Nikiforov YE (2005). Editorial: anaplastic carcinoma of the thyroid--will aurora B light a path for treatment?. J Clin Endocrinol Metab.

[28] Shimaoka K, Schoenfeld DA, DeWys WD, Creech RH, DeConti R (1985). A randomized trial of doxorubicin versus doxorubicin plus cisplatin in patients with advanced thyroid carcinoma. Cancer.

[29] Gottlieb JA, Hill CS (1974). Chemotherapy of thyroid cancer with adriamycin. Experience with 30 patients. N Engl J Med.

[30] Wiseman SM, Masoudi H, Niblock P, Turbin D, Rajput A, Hay J (2007). Anaplastic thyroid carcinoma: expression profile of targets for therapy offers new insights for disease treatment. Ann Surg Oncol.

[31] Wagle N, Grabiner BC, Van Allen EM, Amin-Mansour A, Taylor-Weiner A, Rosenberg M (2014). Response and acquired resistance to everolimus in anaplastic thyroid cancer. N Engl J Med.

[32] Grande E, Capdevila J, Diez JJ, Longo F, Carrato A (2013). A significant response to sunitinib in a patient with anaplastic thyroid carcinoma. J Res Med Sci.

[33] Rosove MH, Peddi PF, Glaspy JA (2013). BRAF V600E inhibition in anaplastic thyroid cancer. N Engl J Med.

[34] Bible KC, Suman VJ, Menefee ME, Smallridge RC, Molina JR, Maples WJ (2012). A multiinstitutional phase 2 trial of pazopanib monotherapy in advanced anaplastic thyroid cancer. J Clin Endocrinol Metab.

[35] Ha HT, Lee JS, Urba S, Koenig RJ, Sisson J, Giordano T (2010). A phase II study of imatinib in patients with advanced anaplastic thyroid cancer. Thyroid.

[36] Pennell NA, Daniels GH, Haddad RI, Ross DS, Evans T, Wirth LJ (2008). A phase II study of gefitinib in patients with advanced thyroid cancer. Thyroid.

[37] Cohen EE, Rosen LS, Vokes EE, Kies MS, Forastiere AA, Worden FP (2008). Axitinib is an active treatment for all histologic subtypes of advanced thyroid cancer: results from a phase II study. J Clin Oncol.

[38] Gupta-Abramson V, Troxel AB, Nellore A, Puttaswamy K, Redlinger M, Ransone K (2008). Phase II trial of sorafenib in advanced thyroid cancer. J Clin Oncol.

[39] Savvides P, Nagaiah G, Lavertu P, Fu P, Wright JJ, Chapman R (2013). Phase II trial of sorafenib in patients with advanced anaplastic carcinoma of the thyroid. Thyroid.

[40] Anonymous ESMO; 2014.

[41] Mooney CJ, Nagaiah G, Fu P, Wasman JK, Cooney MM, Savvides PS (2009). A phase II trial of fosbretabulin in advanced anaplastic thyroid carcinoma and correlation of baseline serum-soluble intracellular adhesion molecule-1 with outcome. Thyroid.

[42] Sosa JA, Elisei R, Jarzab B, Balkissoon J, Lu SP, Bal C (2014). Randomized safety and efficacy study of fosbretabulin with paclitaxel/carboplatin against anaplastic thyroid carcinoma. Thyroid.

[43] Banerjee R, Russo N, Liu M, Basrur V, Bellile E, Palanisamy N (2014). TRIP13 promotes error-prone nonhomologous end joining and induces chemoresistance in head and neck cancer. Nat Commun.

[44] Land SC, Tee AR (2007). Hypoxia-inducible factor 1alpha is regulated by the mammalian target of rapamycin (mTOR) via an mTOR signaling motif. J Biol Chem.

[45] Papewalis C, Wuttke M, Schinner S, Willenberg HS, Baran AM, Scherbaum WA (2009). Role of the novel mTOR inhibitor RAD001 (everolimus) in anaplastic thyroid cancer. Horm Metab Res.

[46] Akar U, Ozpolat B, Mehta K, Fok J, Kondo Y, Lopez-Berestein G (2007). Tissue transglutaminase inhibits autophagy in pancreatic cancer cells. Mol Cancer Res.

[47] Kadoch C, Hargreaves DC, Hodges C, Elias L, Ho L, Ranish J (2013). Proteomic and bioinformatic analysis of mammalian SWI/SNF complexes identifies extensive roles in human malignancy. Nat Genet.

[48] Eid JE, Kung AL, Scully R, Livingston DM (2000). p300 interacts with the nuclear proto-oncoprotein SYT as part of the active control of cell adhesion. Cell.

[49] Gaude E, Frezza C (2014). Defects in mitochondrial metabolism and cancer. Cancer Metab.

[50] Kaelin WG, McKnight SL (2013). Influence of metabolism on epigenetics and disease. Cell.

