# Supplementary material for: The genomic and transcriptomic landscape of anaplastic thyroid cancer: implications for therapy
Source: BMC Cancer. 2015 Dec 18;15:984. doi: 10.1186/s12885-015-1955-9 (PMC4683857; doi:10.1186/s12885-015-1955-9)
Supplement: Additional file 2: — Supplementary file outlining the detailed sequencing methodology and additional figures. (DOCX 434 kb) [file 12885_2015_1955_MOESM2_ESM.docx]

# Supplementary Appendix

# This appendix has been provided by the authors to give readers additional information about their work.

Supplement to: **The Genomic and Transcriptomic Landscape of Anaplastic Thyroid Cancer: Implications for Therapy**

Katayoon Kasaian, Sam M Wiseman, Blair A Walker, Jacqueline E Schein, Yongjun Zhao, Martin Hirst, Richard A Moore, Andrew J Mungall, Marco A Marra, Steven JM Jones

**MATERIALS AND METHODS**

**Library preparation and sequencing**

Tumor biopsy specimen collected from the patient was embedded in Tissue-Tek O.C.T. (optimal cutting temperature) compound (Sakura Finetek USA, Inc.) and sectioned for DNA extraction. Using 1ug DNA each from the tumor and blood and 3 cell lines, THJ-16T, THJ-21T and THJ-29T, five whole genome libraries were constructed using a modified version of Illumina TruSeq PCR free protocol (FC-121-3001). In brief, 1ug genomic DNA was sheared for 45 sec, duty cycle 10%, intensity 5 burst per second 200 using Covaris E210, to an average of 400bp. NEB Paired-End Sample Prep Kit (New England Biolabs, USA) was used in library construction. Following the end repair reaction, a size selection was done using Ampure XP bead (Beckman-Coulter, USA). The sample:bead ratio was 110:27 for upper cut and 137:15 for lower cut, respectively. The resulting size selected fraction, 300-500 bp, was A-tailed, and ligated to Illumina TruSeq adapters. The PCR-free libraries were cleaned up with Ampure XP beads and quantified by qPCR assay using the KAPA SYBR FAST qPCR kit (Kapa Biosystems (Pty) Ltd, South Africa). Paired-end 100 bp reads were generated on Illumina HiSeq2500 instruments following the manufacturer’s protocol with minor variations. Software version HCS1.5.8 was utilized.

For whole transcriptome sequencing, RNA was extracted from 7 cell lines using MACS mRNA isolation kit (Miltenyi Biotec), resulting in 5-10 μg of DNase I-treated total RNA as per the manufacturer’s instructions. Double-stranded cDNA was synthesized from the purified poly(A)^+^ RNA using the Superscript Double-Stranded cDNA Synthesis kit (Invitrogen) and random hexamer primers (Invitrogen) at a concentration of 5 μM. The cDNA was fragmented by sonication and a paired-end sequencing library prepared following the Illumina paired-end library preparation protocol. Cluster generation and sequencing were performed on the Illumina HiSeq instruments following the manufacturer’s recommended protocol, producing 75bp paired-end non-stranded whole transcriptome sequence data. One transcriptome library from the tumor was constructed using 3ug RNA by following the strand specific RNA-Seq protocol [1], with a few modifications. Briefly, PolyA+ RNA was purified using the MultiMACS mRNA isolation kit on the MultiMACS 96 separator (Miltenyi Biotec, Germany). The eluted PolyA+ RNA was ethanol precipitated and re-suspended in 10µL of DEPC treated water. First-strand cDNA was synthesized from the purified polyA+ RNA using the Superscript cDNA Synthesis kit (Life Technologies, USA) and random hexamer primers at a concentration of 5µM along with a final concentration of 1ug/ul Actinomycin D. The second strand cDNA was synthesized following the Superscript cDNA Synthesis protocol by replacing the dTTP with dUTP in dNTP mix, allowing the second strand to be digested by UNG (Uracil-N-Glycosylase, Life Technologies, USA) post adapter ligation to achieve strand specificity. Library construction was carried out following a modified version of the Illumina paired end library protocol using the NEB Paired-End Sample Prep Kit (New England Biolabs, USA), the adapter-ligated products were purified using Ampure XP beads and digested with UNG (1U/ul) at 37°C for 30 min followed by deactivation at 95°C for 15 min. The digested cDNA was purified using Ampure XP beads, and then PCR-amplified with Phusion DNA Polymerase (Thermo Fisher Scientific Inc. USA) using Illumina’s PE primer set, with cycle condition 98˚C 30 sec followed by 10 cycles of 98˚C 10 sec, 65˚C 30 sec and 72˚C 30 sec, and then 72˚C 5min. Paired-end 75bp reads were generated on Illumina HiSeq2500 following the manufacturer’s protocol with minor variations. Software version HCS1.5.8 was utilized.

**FIGURES**

**Figure S1**. Focal copy number variant regions in THJ-21T and THJ-29T cell lines

**Figure S2.** Downregulation of thyroid differentiation marker genes in ATC. Median, first and third quartile values are marked for each distribution.


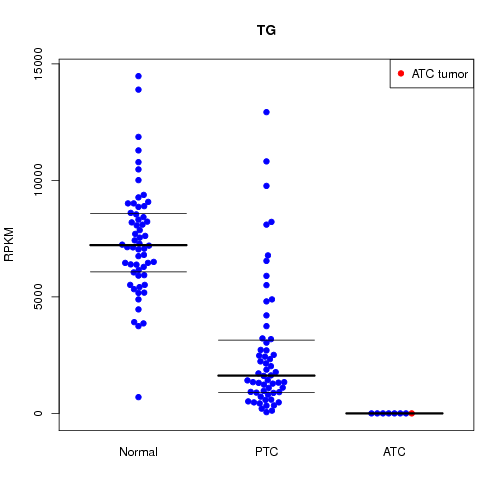

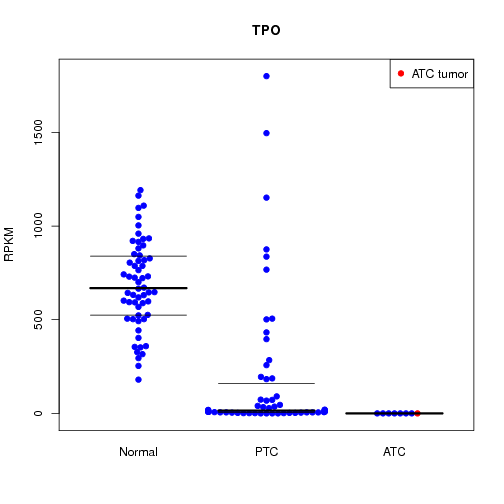

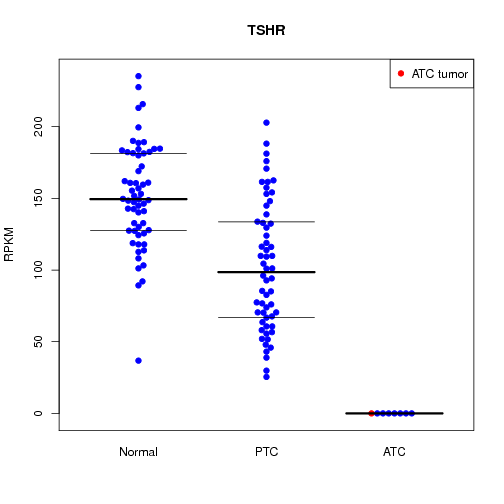

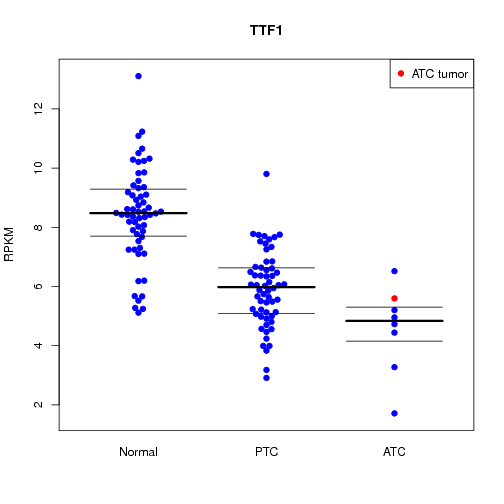


**Figure S3.** Downregulation of potential cancer drivers and drug targets in ATC. Median, first and third quartile values are marked for each distribution


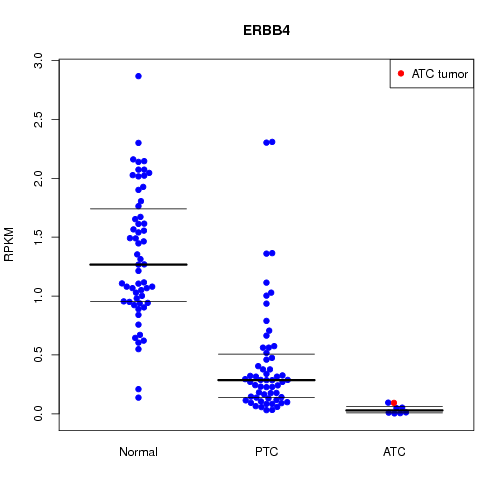

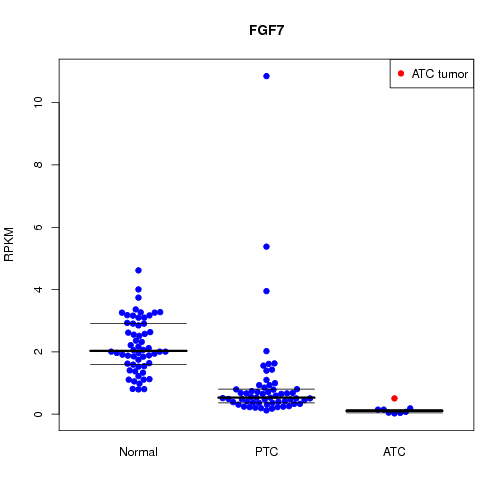

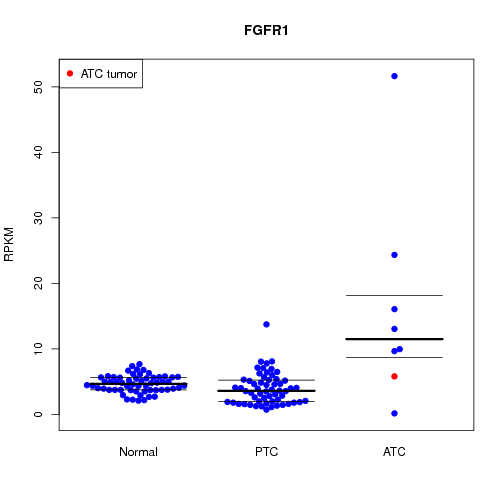

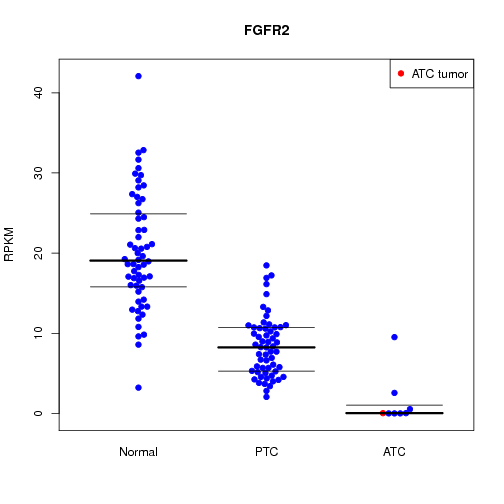

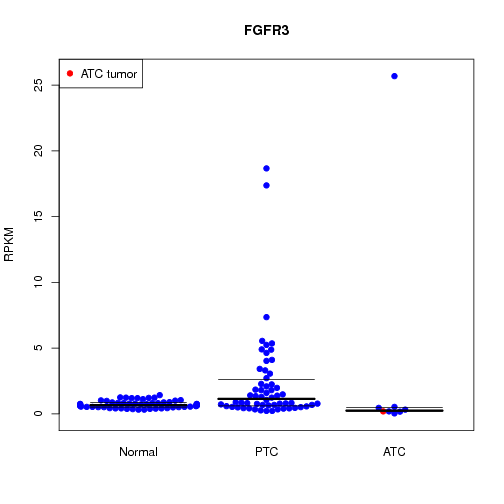

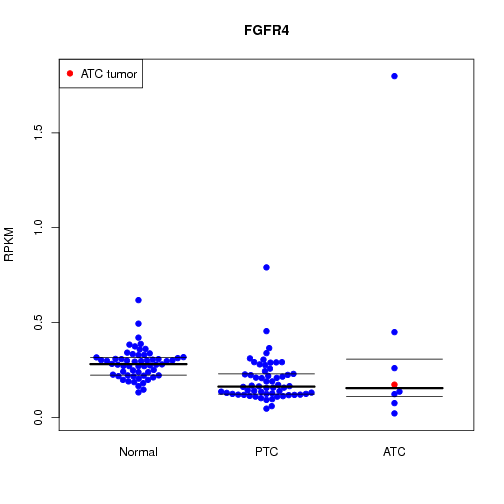

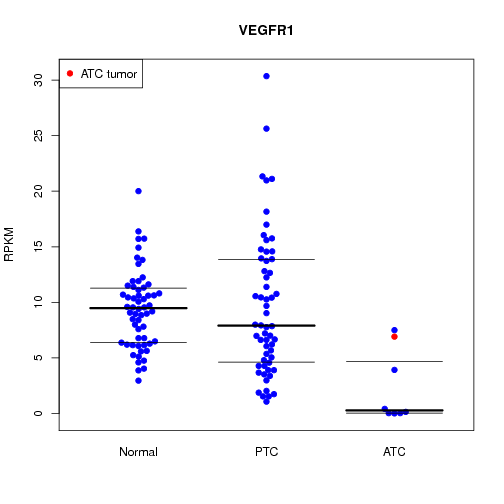

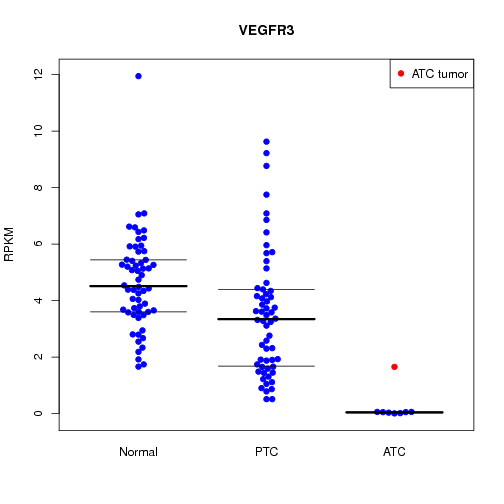

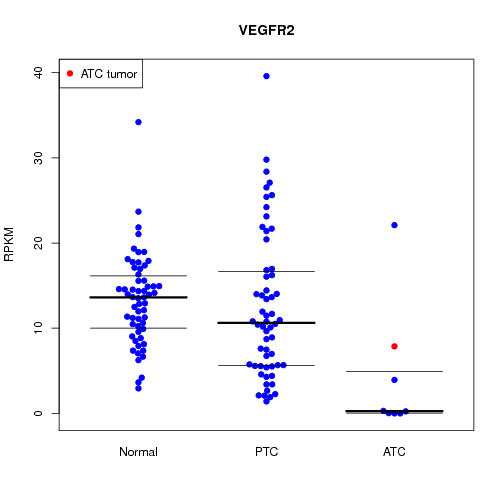

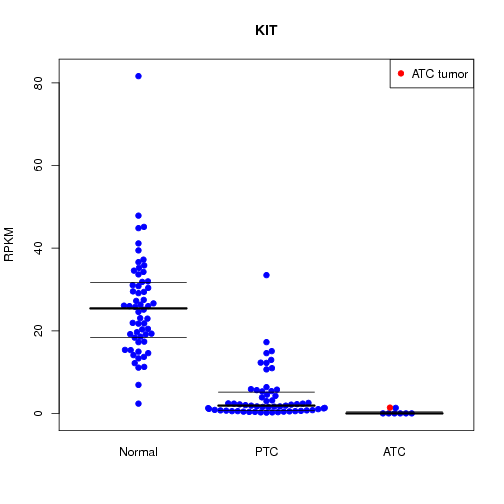

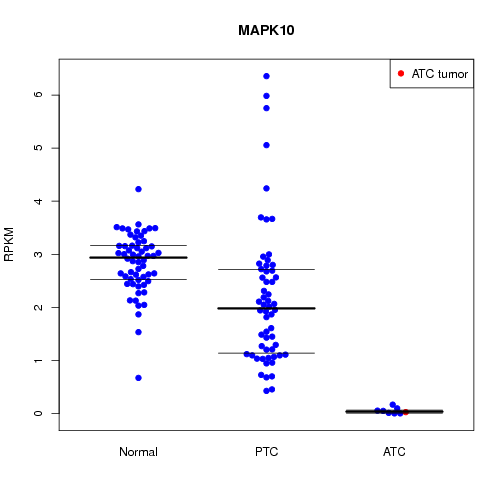

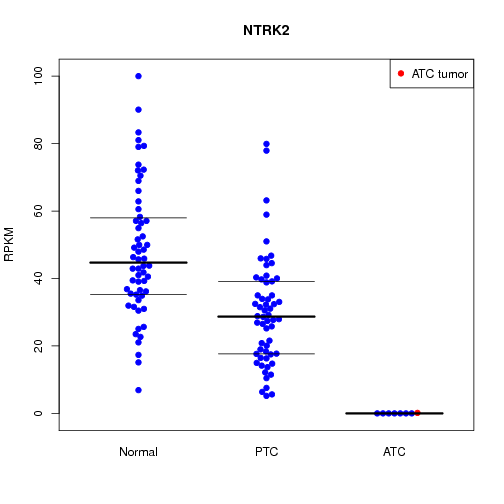

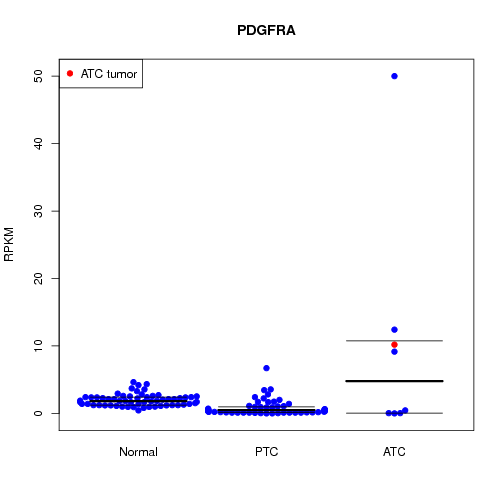

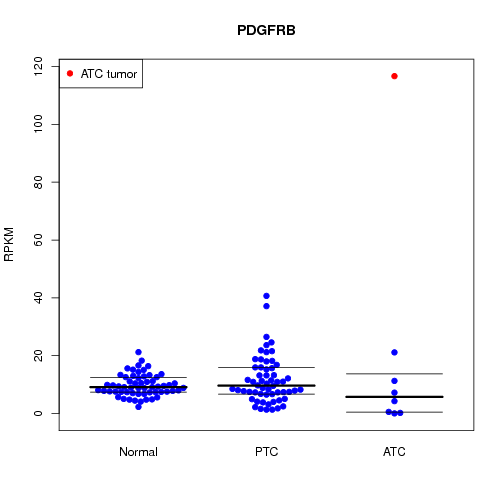

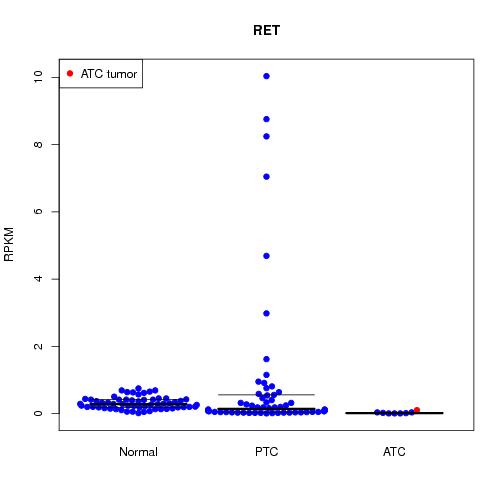

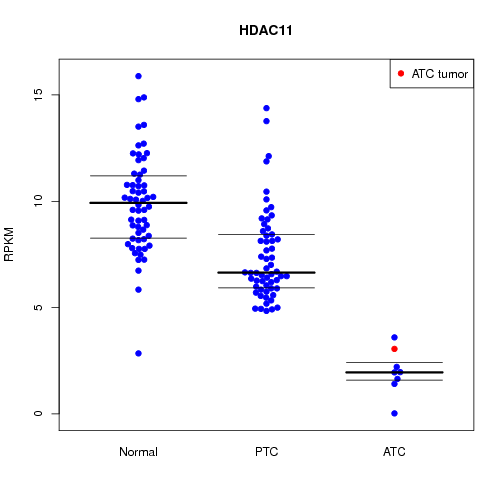

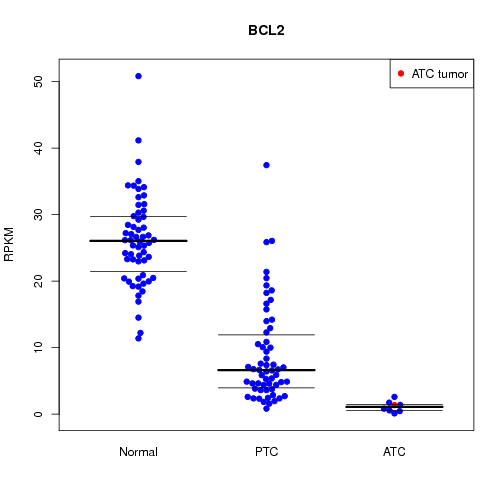

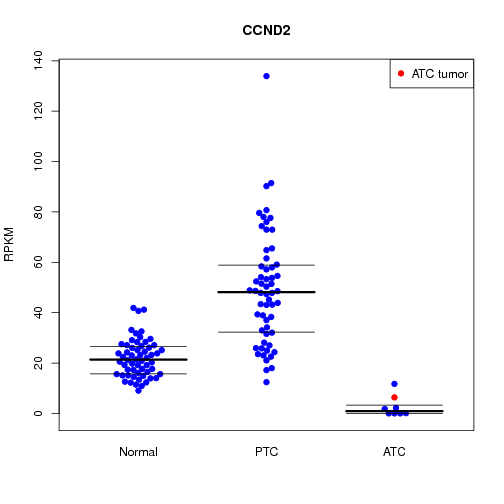


**REFERENCES**

1. Parkhomchuk D, Borodina T, Amstislavskiy V, Banaru M, Hallen L, Krobitsch S, Lehrach H, Soldatov A: **Transcriptome analysis by strand-specific sequencing of complementary DNA.** Nucleic Acids Res 2009, **37**(18):e123.
